# Renoprotective Effects of Incretin-Based Therapy in Diabetes Mellitus

**DOI:** 10.1155/2021/8163153

**Published:** 2021-08-21

**Authors:** Habib Yaribeygi, Stephen L. Atkin, Fabrizio Montecucco, Tannaz Jamialahmadi, Amirhossein Sahebkar

**Affiliations:** ^1^Research Center of Physiology, Semnan University of Medical Sciences, Semnan, Iran; ^2^Weill Cornell Medicine Qatar, Doha, Qatar; ^3^IRCCS Ospedale Policlinico San Martino Genoa-Italian Cardiovascular Network, 10 Largo Benzi, 16132 Genoa, Italy; ^4^First Clinic of Internal Medicine, Department of Internal Medicine, University of Genoa, 6 Viale Benedetto XV, 16132 Genoa, Italy; ^5^Department of Food Science and Technology, Quchan Branch, Islamic Azad University, Quchan, Iran; ^6^Department of Nutrition, Faculty of Medicine, Mashhad University of Medical Sciences, Mashhad, Iran; ^7^Biotechnology Research Center, Pharmaceutical Technology Institute, Mashhad University of Medical Sciences, Mashhad, Iran; ^8^Applied Biomedical Research Center, Mashhad University of Medical Sciences, Mashhad, Iran; ^9^School of Pharmacy, Mashhad University of Medical Sciences, Mashhad, Iran; ^10^Department of Medical Biotechnology, School of Medicine, Mashhad University of Medical Sciences, Mashhad, Iran

## Abstract

Glucagon-like peptide-1 (GLP-1) receptor agonists are recently discovered antidiabetic drugs with potent hypoglycemic effects. Among different mechanisms of activity, these compounds were shown to reduce blood glucose by suppression of glucagon secretion and stimulation of glucose-dependent insulin secretion. These antidiabetic agents have a minor risk of hypoglycemia and have been suggested as a second-line therapy to be added to metformin treatment to further optimize glycemic control in diabetes. More recently, scientific evidence suggests that GLP-1 receptor agonists may particularly afford protection from diabetic nephropathy through modulation of the molecular pathways involved in renal impairment and so improve renal function. This additional benefit adds further weight for these compounds to become promising drugs not only for glycemic control but also to prevent diabetic complications. In this review, we have updated evidence on the beneficial effects of GLP-1 receptor agonists on diabetic nephropathy and detailed the underlying pathophysiological mechanisms.

## 1. Introduction

The global prevalence of all types of diabetes mellitus (DM) is increasing [1]. This metabolic disorder is accompanied by not only deteriorating metabolism of glucose but also lipids and amino acids [1, 2]. On the other hand, DM is also associated with other pathophysiologic molecular mechanisms including oxidative stress, inflammatory responses, and apoptosis. This results in aberrant metabolic pathways, involving polyol, hexokinases activation, cell death receptor activity, modulation of different transcription factors and protein kinases, and other molecular mechanisms such as renin-angiotensin system (RAS) activation and autonomic nervous system (ANS) effects [3]. Therefore, all diabetic complications including the microvascular diabetic complications of diabetic nephropathy (DN), diabetic retinopathy (DR) and diabetic neuropathy, and macrovascular disease such as cardiovascular disorders have been recently hypothesized to be caused by a combination of abnormal glucose levels and the above-cited mechanisms [4].

Glucagon-like peptide-1 receptor (GLP-1R) agonists are novel antidiabetic drugs that have potent hypoglycemic effects and reduce blood glucose by suppression of glucagon secretion and through glucose-dependent insulin secretion [5, 6]. These antidiabetic agents have a minor risk of hypoglycemia and have been suggested as second-line therapy to be added to metformin treatment to optimize glycemic control in diabetes [7]. Alternatively, dipeptidyl peptidase-4 inhibitors (DPP-4i) may be used to prevent the degradation of endogenous GLP-1 leading to its prolonged activity [8].

There is now recent evidence suggesting that these agents may prevent DN [9–11]. In this review, we have presented the evidence for the beneficial effect of glucagon-like peptide-1 receptor agonists on diabetic nephropathy and detailed the underlying molecular mechanisms involved in its pathophysiology. These agents may provide further protection of the kidney by indirect and “pleiotropic” effects in addition to optimizing glycemic control [12].

## 2. Glucagon-Like Peptide Receptors and Related Analogues

Glucagon-like peptide-1 (GLP-1) is a protein belonging to the incretin family that is released from the intestine in response to food ingestion [13]. GLP-1R is found on pancreatic islets' beta cells and is involved in the control of glycemia by induction of insulin release and suppression of glucagon secretion [14]. In humans, it is synthesized by the GLP-1R gene that is located on chromosome 6 [15, 16]. GLP-1R is a member of the glucagon receptor family of G protein-coupled receptors that are composed of two domains, an extracellular domain that binds the C-terminal helix of GLP-1, and a transmembrane domain that binds to the N-terminal region of GLP-1 [17–20]. Incretin therapy for diabetes control is based on GLP-1R agonists and DPP-4i whose glycemic effects are mediated by these receptors (Table 1) [5, 21]. GLP-1R agonists are a class of recently introduced antidiabetic drugs that were approved by the FDA in 2010 for the treatment of diabetic patients [21]. The incretin family includes intestinal GLP-1 and gastric inhibitory peptide (GIP), which reduce postprandial blood glucose by inhibition of glucagon secretion from pancreatic *α*-cells and stimulate insulin release from *β*-cell in a glucose-dependent manner [22–24]. Moreover, they can have additional effects, such as delaying gastric emptying, suppression of appetite, decreased nutrient absorption from the gut, improvement of lipid metabolism, and inhibition of pancreatic *β*-cell apoptosis [23, 25, 26]. GLP-1R activation is followed by increased production of cAMP (cyclic adenosine monophosphate), cellular depolarization, and an increase in intracellular calcium concentration leading to insulin secretion from pancreatic *β*-cells [24, 27].

DPP-4 is a ubiquitous enzyme that is widely distributed [28]. DPP-4 inhibitors have their hypoglycemic effect by preventing endogenous GLP-1 inactivation leading to glucose-dependent insulin release from islet *β*-cells and glucagon release suppression [8]. After posttranslational processing of preglucagon (PG) peptides in the intestinal L cells, at least four separate forms of PG are secreted that all can be inactivated by DPP-4 by removing the two amino acids from the N-terminal residue [29]. Therefore, DPP-4i have a similar hypoglycemic mechanism to GLP-1 agonists, although GLP-1 agonists have been hypothesized to affect additional pleiotropic effects, may reduce body weight, and improve metabolic variables [8].

## 3. Molecular Mechanisms Involved in the Pathophysiology of DN

Renal dysfunction in diabetic patients is common with a high prevalence of up to 40 percent and underlies the development of end-stage renal disease (ESRD) leading to complete renal failure requiring hemodialysis [30, 31]. DN pathophysiology includes alteration of several molecular pathways, such as oxidative damage, inflammatory responses, apoptosis, protein kinase c (PKC), renin-angiotensin system (RAS) activation, adenosine, toll-like receptor (TLR) activation, transforming growth factor-*β* (TGF-*β*), nitric oxide (NO) synthesis, tumor necrosis factor-*α* (TNF-*α*), death receptors, JAK/STAT (Janus kinase/signal transducers and activators of transcription) pathway, and different types of adhesion molecules [3, 32]. Agents that can modulate these pathways may provide beneficial effects for improving renal function [2, 21, 33].

## 4. Renoprotective Effects of GLP-1R Agonists

**Figure 1:**
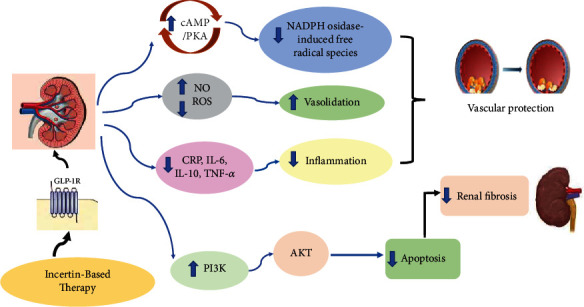
The mechanism underlying the renoprotective effects of incretin therapy (GLP-1RA and DPP-4i).

GLP-1R induction has been reported to protect renal tissue and improve kidney function from diabetes-induced dysfunction via several molecular mechanisms [9–11] that have been detailed in the following paragraphs (Figure 1).

### 4.1. Antioxidant Activity

DM has been associated with higher levels of oxidants and free radical production in different organs including renal tissue [21, 34, 35]. There is increasing evidence indicating that GLP-1R induction might attenuate oxidative markers and ameliorate oxidative damage [36–39]. Wu and colleagues in 2011 demonstrated that treatment with the GLP-1R agonist exenatide decreased oxidative stress markers in diabetic patients [38]. Bunck and coworkers in 2010 reported that 51-week therapy with exenatide was associated with significant reduction in oxidative stress markers of malondialdehyde (MDA) and oxidized low-density lipoprotein (oxLDL) in diabetic patients [36]. The authors suggested that the antioxidative effects of this drug might be related to the lowering of postprandial glucose [36].

Chang and colleagues in 2013 reported that exenatide therapy markedly reduces IR- (ischemia-reperfusion-) induced oxidative stress in both *in vitro* and *in vivo* models [37]. The authors showed that exenatide was able to directly inhibit H_2_O_2_-induced free radical species production and decrease the levels of lactate dehydrogenase (LDH), creatine kinase-MB (CK-MB), and MDA, whilst potentiating the antioxidant defense system elements of superoxide dismutase (SOD) [37]. Moreover, Holz in 2004 suggested that GLP-1R induction led to its antioxidant effects by cAMP- (cyclic adenosine monophosphate-) dependent pathways [40]. Similarly, there is evidence for comparable effects of DPP-4i [11, 41, 42]. Pujadas and coworkers reported that the DPP-4i teneligliptin acts as a potent antioxidant agent, potentiating the antioxidant defense system and reducing NADPH (nicotinamide adenine dinucleotide phosphate) oxidase-induced free radical species, reducing oxidative damage in human umbilical vein endothelial cells [41].

On the other hand, GLP-1R induction can be achieved via the NRF2 (nuclear factor [erythroid-derived 2]-like 2) signaling pathway [13, 41]. NRF2 is a main regulator of gene transcription of antioxidant elements, and its activation is correlated to protection against oxidative damage [13]. Oh and Jun in 2017 concluded that GLP-1RA was able to induce NRF2 activation and protect against oxidative stress [13]. Accordingly, Kim et al. reported that exendin-4 therapy activates NRF2 signaling pathways and inhibits oxidative damage in pancreatic tissues [43].

There is also direct evidence in kidney tissues of a GLP-1-mediated antioxidant activity [44]. Civantos et al. in 2017 showed that the DPP-4i sitagliptin reduces oxidative stress in renal tissues by the NRF2-dependent pathway in an experimental model of DN [44]. Also, Yang et al. in 2020 found that liraglutide prevented diabetic kidney disease through several pathways as well as reversed NRF2 translocation into the nuclei whilst suppressing oxidative stress in diabetic obese rats [45]. Moreover, Liang et al. in 2020 provided further direct evidences indicating liraglutide protects against diabetic nephropathy by downregulation of TXNIP (thioredoxin-interacting protein), which acts as an oxidative stress inducer in male high fed diet mice [46]. Similarly, Wang and coworkers reported that exenatide exerts renoprotective effects by suppressing oxidative damage in diabetic rats [47]. Therefore, it has been hypothesized that improvement of renal function and reduction in albuminuria might be associated with the antioxidant properties of these agents [48–50].

### 4.2. Anti-Inflammatory Activity

Inflammation has a pivotal role in DN pathophysiology [21, 51, 52], and there is evidence that GLP-1 may have anti-inflammatory activities [53, 54]. Wu and coworkers in 2011 found that treatment with exenatide attenuated serum levels of inflammatory molecules, such as monocyte chemoattractant protein-1 (MCP-1) and high-sensitivity C-reactive protein (hs-CRP), leading to lower grade inflammation in diabetic subjects [38]. In addition, Satoh-Asahara and colleagues in 2013 demonstrated that sitagliptin markedly decreased the serum levels of CRP, interleukin- (IL-) 6, IL-10, and tumor necrosis factor- (TNF-) *α* in diabetic patients [54]. Moreover, Marques and coworkers in 2014 demonstrated that sitagliptin therapy attenuated the levels of IL-1*β* and TNF-*α* and decreased the inflammatory responses in kidneys in diabetic animals [55]. Similarly, Kodera and coworkers in 2011 suggested that the renoprotective effects of GLP-1 are independent of its hypoglycemic effects and that it is completely related to its anti-inflammatory activity [56]. More recently, Cappetta and colleagues showed that DPP-4 inhibition might reduce the progression of DN by suppressing the inflammatory mediators, such as TNF-*α*, IL-1*β*, IL-6, and MCP-1 in rats [57], suggesting a potential immunomodulatory effect for these drugs. In addition, Wang and colleagues in 2019 reported that exenatide provided protective effects against DN via alleviating the inflammatory responses in diabetic rats [47]. They found that exenatide reduced inflammatory markers such as TNF-*α*, IL-6, CRP, and CCL-5 (chemokine C-C motif ligand 5) in renal tissues [47]. Moreover, Muskiet et al. in an extensive clinical study demonstrated renoprotective effects of incretins and suggested that they provide these beneficial effects via anti-inflammatory properties [58]. These data strongly suggest that GLP-1 action might have direct renoprotective effects by at least partly suppressing inflammation [39, 54–56].

### 4.3. Vascular Protection

The level of nitric oxide (NO) bioavailability has a key role in the normal function of vascular endothelium as well as the normal-physiologic hemodynamic state [59]. In DN, NO bioavailability is reduced [33, 59]. Some studies have shown the renoprotective effects of agents via modulating NO synthesis and/or NOS (nitric oxide synthase) expression [60, 61].

Emerging evidence suggests that GLP-1 can modulate NO synthesis [62, 63]. Barale and colleagues in 2017 reported that GLP-1 was able to modify NO bioavailability and activity in platelets [62]. Sélley and coworkers in 2014 demonstrated that exenatide decreased blood pressure and caused vasodilation in a dose-dependent manner by producing NO [64]. This NO modulating effect may be protective for the development of renal failure in diabetes [65]. Thomson and coworkers in 2017 demonstrated that exenatide modified the renal hemodynamic state and improved the glomerular filtration rate (GFR), providing renoprotective effects by a mechanism dependent on NO synthesis and bioavailability [65].

GLP-1 receptors are highly expressed in renal vessels and therefore play an important role in their function [66, 67]. Jensen et al. in 2020 found that renal vasodilation is closely dependent on GLP-1 receptors in kidneys indicating that these receptors have significant roles in renal vasculature homeostasis and kidney function [67]. They observed that hypertensive animals with kidney dysfunction had reduced GLP-1 receptors in their renal tissues. Cappetta and colleagues in 2019 proposed that DPP-4 inhibition might protect animals from the development of renal failure by inducing endothelial nitric oxide synthase (eNOS) enzyme activity and improving endothelial function [57]. Moreover, Filippatos and Elisaf in 2013 reported that GLP-1 improved renal function modulating NO and regulating renal vasculature function [68]. More recently, Bjørnholm et al. in 2021 found that GLP-1 agonists have regulatory effects on the renal renin-angiotensin system and, thereby, provide modulatory effects on renal vascular [66]. These results were also confirmed in other reports [69], indicating that GLP-1 could improve renal function by modulating NO bioavailability and promoting vascular endothelial function.

### 4.4. Antiapoptotic and Antifibrotic Effects

GLP-1 may ameliorate DM-induced kidney injury by suppressing renal cell apoptosis and fibrosis that are increased under chronic hyperglycemia [37, 70]. In 2013, Chang and colleagues found that exenatide suppressed apoptotic pathways through PI3K/Akt (phosphoinositide 3-kinases/protein kinase B) signaling pathways [37]. Also, Pujadas and coworkers reported that the DPP-4i teneligliptin ameliorated diabetes-induced kidney tissue injury by inhibiting apoptotic pathways in human endothelial cells [41]. Moreover, Tews and colleagues in 2009 demonstrated that exendin-4 decreased cytokine-induced tissue injury by abrogating the apoptotic pathways in pancreatic beta cells [71]. Furthermore, Marques et al. in 2014 showed that GLP-1 through sitagliptin decreased apoptotic cell death and improved kidney function in diabetic animals [55].

The fibrotic process is induced by various stimuli such as free radical species and inflammatory cytokines. It plays a significant role in diabetes-induced tissue injury as well as in DN [72]. Modulating fibrosis is an attractive therapeutic option [73, 74]. GLP-1 might modulate the fibrotic process and so reduce tissue injury [75]. In 2016, Shi et al. showed that DPP-4i might have renoprotective effects impacting on fibrotic cells [75]. Furthermore, Shi and coworkers in 2014 showed that linagliptin ameliorates diabetes-mediated kidney injury by lowering the rate of fibrotic processes in diabetic mice [75]. Li et al. showed that liraglutide inhibited the fibrotic process in the rat renal tubular epithelial cell line (NRK-52E) [76]. The authors suggested that liraglutide might have an impact on the renal fibrosis by reducing the activation of TGF-*β*1/Smad3 (a main mediator of fibrosis) and ERK1/2 (extracellular signal-regulated kinases) signaling pathways [76]. Also, Xu et al. showed that the DPP-4i sitagliptin improved renal function via inhibition of renal fibrosis by upregulating the survival factor of glucose-regulated protein 78 (GRP78) and downregulating the TGF-*β*1 in a dose-dependent manner [77]. Moreover, Liang et al. in 2020 found that liraglutide provided renoprotective effects by ameliorating apoptosis in diabetic mice [46]. These data suggest that GLP-1 activity can ameliorate kidney injury by suppressing cellular death via apoptotic and fibrotic processes.

## 5. Conclusion

DN is the most common cause of kidney dysfunction leading to end-stage renal failure requiring hemodialysis. Optimal diabetes control through therapeutic interventions may lower the risk of diabetic complications. Novel hypoglycemic agents of GLP-1R agonists and DPP-4i can provide renoprotective effects beyond their glucose-lowering effects. Their activities are mediated through GLP-1R induction mainly located in pancreatic islets' beta cells. The data strongly suggests that increased GLP-1 activity via administration of GLP-1R agonists or DPP-4i may reduce main molecular mechanisms underlying DN, such as oxidative stress, inflammation, endothelial function, and cellular death dependent on apoptosis and/or fibrosis (Table 2). Additional clinical studies are still needed to clarify the potential glucose-independent beneficial effects of GLP-1 agonists in human DN.

## Figures and Tables

**Table 1:** GLP-1RA and DPP-4i families.

Class of drugs	Approved forms	Effects	Ref.
GLP-1RA	Exenatide, liraglutide, albiglutide, semaglutide, lixisenatide, and dulaglutide	Directly induce GLP-1R activity and mimetic incretin hormone hypoglycemic effects	[22]
DPP-4i	Sitagliptin, vildagliptin, saxagliptin, linagliptin	Block the DPP-4 enzyme activity and thereby increase the active levels of GLP-1	[8]

**Table 2:** Main molecular mechanisms by which GLP-1 activity affords renoprotective effects.

Molecular mechanisms	Effects on renal function	Ref.
Antioxidant activity	Decrease the levels of LDH, oxLDL, CK-MB, and MDA; induces NRF2 activity and potentiates antioxidant defense system; decreases NOX activity and free radical production, ameliorates oxidative damage	[11, 13, 31, 41, 42]
Anti-inflammatory activity	Decrease the inflammatory mediators of NF-*κ*B, hs-CRP, TNF-*α*, IL-1*β*, IL-6, and MCP-1 leading to a lower rate of inflammation	[38, 54–56]
Vascular endothelial function	Induces eNOS activity and improves vascular endothelium function	[57, 60–64, 68, 69]
Antiapoptotic and antifibrotic effects	Inhibits molecular pathways upstream to apoptosis and fibrosis by various pathways such as TGF-*β*1/Smad3 and ERK1/2 signaling pathways and so protects tissues against cellular death	[37, 41, 71, 72, 75–77]

LDH = lactate dehydrogenase; CK-MB = creatine kinase-MB; MDA = malondialdehyde; NF-*κ*B = nuclear factor kappa-B; MCP-1 = monocyte chemoattractant protein-1; IL = interleukin; TNF-*α* = tumor necrosis factor-*α*; NRF2 = nuclear factor (erythroid-derived 2-) like 2; hs-CRP = high-sensitivity C-reactive protein; eNOS = endothelial nitric oxide synthase; Smad3 = a main mediator of fibrosis; ERK1/2 = extracellular signal-regulated kinases.
